# Supplementary material for: Genomic Investigation of Bacterial Co-Infection in Southern Pudu (Pudu puda) with Fatal Outcome: Application of Forensic Microbiology in Wildlife Impacted by Anthropogenic Disasters
Source: Animals (Basel). 2025 Aug 20;15(16):2435. doi: 10.3390/ani15162435 (PMC12382652; doi:10.3390/ani15162435)
Supplement: Supplementary file 1 [file animals-15-02435-s001.zip › animals-3783156-supplementary/Supplementary Table S1.pdf]

Supplementary Table S1. Quality control and genomic characteristics of strains isolated from the southern pudu (*Pudu puda*).

Characteristics	Strains			
	**MVL-11-23**	**MVL-123-23**	**MVL-12-23**	**MVL-13-23**
Completeness	99.97	99.97	100	100
Contamination	0.31	0.22	0.70	0.00
No. of Contigs	72	78	111	17
No. of Contigs (> = 0 bp)	84	85	113	17
No. of Contigs (> = 1000 bp)	62	66	101	16
Largest contig	542568	403442	414366	1400539
Total length	4918802	4855690	6146375	3791095
Total length (> = 0 bp)	4924047	4858718	6147252	3791095
Total length (> = 1000bp)	4911121	4846168	6139629	3790525
N50	228455	204583	132092	367380
N90	68501	68499	42358	204213
L50	8	9	15	3
L90	22	24	44	7
GC (%)	50.94	50.93	54.82	38.08
**Per base quality**				
No. of N's per 100kbp	0	0	0	0
No. of N's	0	0	0	0
