# Supplementary material for: Genomic Investigation of Bacterial Co-Infection in Southern Pudu (Pudu puda) with Fatal Outcome: Application of Forensic Microbiology in Wildlife Impacted by Anthropogenic Disasters
Source: Animals (Basel). 2025 Aug 20;15(16):2435. doi: 10.3390/ani15162435 (PMC12382652; doi:10.3390/ani15162435)
Supplement: Supplementary file 1 [file animals-15-02435-s001.zip › animals-3783156-supplementary/Supplementary Table S4.pdf]

Supplementary Table S4. Virulence genes (virulome) according to the virulence factor class of *Klebsiella oxytoca*, *Escherichia coli*, and *Acinetobacter baumannii* strains isolated from southern pudu.

**Virulence factor class**	***K. oxytoca*** **MVL-12-13**	***E. coli*** **MVL-11-23**	***E. coli*** **MVL-123-23**	***A. baumannii*** **MVL-13-23**
**Adherence**	*bcfABDEFG, mrkABCDFHIJ, fimABCDEFGHIK, cfaBC, ecpAR, papCD, pilW, stbABCDE, stiB*	*cfaABC, ecpABCDE, elfACDG, eaeH, hcpABC, fimABCDEFGHI, flgC, pegBC, stcA*	*cfaABC, cfaD/cfaE, cgsDEFG, csgA, ecpABCDE, elfACDG, eaeH, focC, hcpABC, papC, fimABCDEFHI, fleR, fliQ, htpB, flgCI, lfgGH, lfhA, lfiI, flmH, nueA, plr/gapA, pilT, pilW, pegBC, staBC, stcABCD, stfCD, stgAB*	*ompA, pilA*
**Aminoacid and purine metabolism**			*glnA1*	
**Anaerobic respiration**			*narH*	
**Antiphagocytosis**			*algU, rmlB, wbjD/wecB, wecC, galF, wcal, wzbc*	
**Autotransporter**		*agn43, cah, ehaB, upaG/ehaG*	*agn43, cah, ehaB, upaG/ehaG*	
**Biofilm formation**				*adeFGH, bap, csuA/B, csuABCDE, pgaABCD*
**Cell surface components**	*sugC*		*sugC*	
**Chemootaxis and motility**			*fliP*	
**Efflux pump**	*acrAB*		*acrB, farB*	
**Endotoxin**			*gmhA/lpcA, htrB, kdsA, kdtA, lpxABCDK, msbA, opsX/rfaC, orfM, rfaDEF, wecA*	
**Enzyme**			*eno*	*plc, plcD*
**Invasion**		*ibeBC*	*ibeBC, cheBRWYZ, motA*	
**Immune evasion**			*galEU, mrsA/glmM, pgi, acpXL*	*lpsB, lpxABCDLM*
**Iron uptake**	iutA, *entABCDEFS, fepABCDG, fes, iroEN, fyuA, irp1, irp2, ybtAEPQSTUX*	*fyuA, irp1, irp2, ybtAEPQSTUX*	*iroN, fyuA, irp1, irp2, ybtAEPQSTUX, ccmF, entABCDEF, fepBCDG, hemCEHLN*	*barAB, basABCDFGHIJ, bauABCDEF, entE*
**Lipid and fatty acid metabolism**			*icl, panD*	
**Magnesium uptake**	*mgtB*		*mgtB*	
**Motility**			*motB*	
**Non-LEE encoded TTSS effectors**		*espL1, espL4, espR1, espX1, espX4, espX5*	*espL1, espL4, espR1, espX1, espX4, espX5*	
**Nutritional factor**	*allABCDRS*			

**Nutritional virulence**			*bioB*	
**Protease**	*pla*			
**Secretion system**	clpV/*tssH, hcp/tssD, icmF/tssM, impA/tssA, sciN/tssJ, tle1, tssFG, vasE/tssK, vgrG/tssI,vipB/tssC clpV, dotU, ompA, impF*	*clpV1*	*epsE, flgBCDEFGHIJKLM, flhABCD, fliACEFGHIJLMNPQRSZ, clpV1, ipaH, gspCDEFGHIJKLM, exeDG, clpB, spaQ*	
**Serum resistance**			*rmlA*	*pbpG*
**Stress adaptation**	*katA*		*katG, sodCI*	*katA*
**Toxin**		*hlyE/clyA*	*hlyE/clyA, hlyA, cysC1*	
**Quorum sensing**			*luxS*	
**Virulence regulation**	*rcsAB, phoP*		*rpoS, csrA, gacA, phoPQ, rcsA*	*abaIR, bfmRS*
**Others**		*wcaG*	*msbB2, wcaG*	
