# Supplementary figures and images for: Genomic Investigation of Bacterial Co-Infection in Southern Pudu (Pudu puda) with Fatal Outcome: Application of Forensic Microbiology in Wildlife Impacted by Anthropogenic Disasters
Source: Animals (Basel). 2025 Aug 20;15(16):2435. doi: 10.3390/ani15162435 (PMC12382652; doi:10.3390/ani15162435)

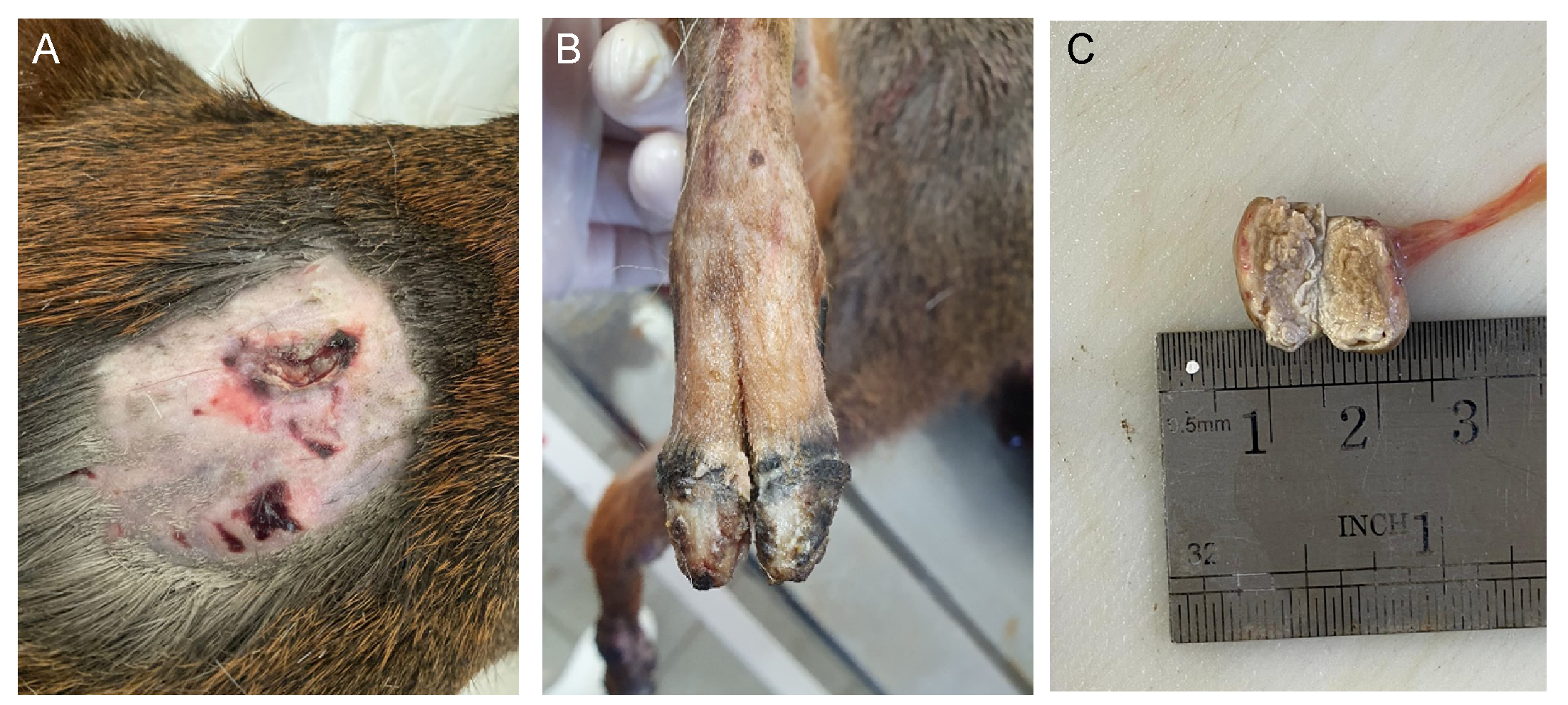

Supplement: Supplementary file 1 [file animals-15-02435-s001.zip › animals-3783156-supplementary/Figure S1.tif]
